# A Double-Gene Metabarcoding Approach for the Authentication of Shrimp Surimi-Based Products

**DOI:** 10.3390/genes16020144

**Published:** 2025-01-24

**Authors:** Jiajie Hu, Alice Giusti, Jixiang Zhang, Lara Tinacci, Chenyang Zhao, Xiaoguo Ying, Andrea Armani, Alessandra Guidi, Shanggui Deng

**Affiliations:** 1 School of Food and Pharmacy, Zhejiang Ocean University, Zhoushan 316022, China; hjj19970214@foxmail.com (J.H.); z806592291@163.com (J.Z.); 18868014254@163.com (C.Z.); dengshanggui@163.com (S.D.); 2 FishLab, Department of Veterinary Sciences, University of Pisa, 56124 Pisa, Italy; alice.giusti@unipi.it (A.G.); lara.tinacci@unipi.it (L.T.); alessandra.guidi@unipi.it (A.G.)

**Keywords:** shrimp surimi, food fraud, food authentication, metabarcoding

## Abstract

Background/Objectives: Shrimp surimi-based products (SSPs) are composed of minced shrimp meat and are highly susceptible to food fraud as fish surimi. This study employed a double-gene metabarcoding approach to authenticate SSPs sold on Chinese e-commerce platforms. Methods: 16S rRNA and 12S rRNA genes were amplified and sequenced from 24 SSPs. Mislabeling was evaluated based on the correspondence between the ingredients (only those of animal origin) reported on the products’ labels and the molecular results. Results: Overall, 87.50% of SSPs (21/24) were found to be mislabeled. The replacement of *Penaeus vannamei* with other shrimp species was particularly noteworthy. Interestingly, in some SSPs, the primary species detected in terms of sequence abundance were not shrimp but fish, pork, chicken, and cephalopods, raising concerns regarding both health risks and ethical issues related to SSP consumption. The 12S rRNA sequencing results revealed that fish species like *Gadus chalcogrammus*, *Evynnis tumifrons*, and *Priacanthus arenatus* were added to some SSPs in significant proportions, with certain products relying on fish priced from “Low” to “High” levels to substitute higher-cost shrimp. Notably, many fish species in SSPs were highly vulnerable to fishing, raising sustainability concerns. Overall, the high mislabeling rate in SSPs, as well as the detection of endangered fish species (*Pangasianodon hypophthalmus*), underscores significant quality control issues. Conclusions: DNA metabarcoding has proven to be an effective tool for ingredient authentication in processed seafood.

## 1. Introduction

The seafood industry has one of the most complex and diverse supply chains within the food sector, which presents significant challenges in maintaining product integrity, especially as demand increases and resources become scarcer [1]. According to the last report of the Food and Agricultural Organization (FAO), China accounts for over one-third (36%) of global aquatic food consumption [2]. As consumer demand continues to grow, China is increasingly adopting advanced value-added processing techniques in place of traditional preservation methods. In 2023, China’s total production of processed aquatic products reached 21,994,645 tons, and surimi-based products, consisting of myofibrillar fish proteins refined through washing minced muscle, alone covered 6.11% of this amount [3,4].

Surimi-based products made from shrimp meat, sometimes reported as shrimp paste (Chinese common name Xia-Hua/虾滑), is especially popular in hot pot cuisine across Asia due to its tender texture [5]. Shrimp species used in these products are especially Pacific white shrimp (*P. vannamei*), Oriental river prawn (*Macrobrachium nipponense*), giant river prawn (*Macrobrachium rosenbergii*), and giant tiger prawn (*Penaeus monodon*) [3]. Besides shrimp, ingredients such as fish, pork, chicken, egg white, salt, starch, and plant protein powder can be included, as in classic surimi [6,7].

Surimi-based products are particularly vulnerable to food fraud motivated by economic gains, especially mislabeling (i.e., false claims or distortion of the information reported on the label), due to the high degree of processing, which makes it more difficult to identify food ingredients through morphological analysis. This illicit practice especially occurs if products are purchased online, where consumers lack the opportunity to closely inspect food labels and are therefore more easily misled by merchants’ promotional claims [8]. Mislabeling, other than undermining consumers’ informed choices, may compromise the sustainability of fishery resources and raise concerns related to food allergies, vegetarianism, religious beliefs, and ethical considerations [9]. Moreover, economic losses for producers are a significant concern, as mislabeling not only results in consumer dissatisfaction but also potential legal consequences and damage to brand reputation [10]. Regulatory challenges also arise, as current food safety laws and guidelines often struggle to enforce accurate labeling in highly processed foods such as surimi, which require more robust testing methods for verification [11].

To date, a number of studies have investigated the composition of surimi-based products (see Table 1 in our previous research [7]), and cases of mislabeling have often been highlighted. The use of vulnerable species and toxic species, as well as the undeclared presence of mollusks, posing a potential health threat for allergic consumers, have also been observed [7,12,13,14,15]. With respect to shrimp, the main ingredient used in shrimp surimi-based products, the replacement of highly valued premium species with lower-quality, lower-priced species was reported [16]. Recently, Kim and Kang [17] observed a 70% mislabeling rate in various commercial products made of shrimp (not including surimi) sold in South Korean markets.

DNA-based methods, especially DNA barcoding, are the most applied methods to detect mislabeling in seafood [9], and their use for this purpose is even recommended by regulations in China [18], the United States [19], and the European Union [20]. However, in the case of highly processed mixed foods such as surimi-based products, the DNA barcoding approach presents limitations [9,21]. This is because it relies on Sanger sequencing, a low-throughput method that produces a partial output, showing only one species at a time (generally the most represented in a sample) and failing to identify the others [22].

In recent years, next-generation sequencing (NGS) technologies have emerged as an efficient detection method capable of obtaining detailed DNA information from mixed food products [9,23]. DNA metabarcoding, which combines NGS with DNA barcoding, can in fact generate amplicons through preliminary PCR and perform rapid and large-scale parallel sequencing. When applied to multi-species products, it can therefore determine the composition, albeit in a semi-quantitative way [9]. With respect to surimi-based products, a recent study applying both DNA barcoding and metabarcoding to authenticate fish cakes (a type of Chinese surimi-based product) highlighted that metabarcoding allowed for the detection of many other undeclared species that had remained hidden using DNA barcoding, including potentially allergenic species such as cephalopods [7].

Metabarcoding can rely on multi-target approach, in which two or more genes are amplified and sequenced. It is widely known that using a multi-target approach provides better estimates of biodiversity and improves taxonomic resolution [24,25,26]. However, a recent systematic review addressing the use of metabarcoding for the authentication of foodstuffs of animal origin highlighted that this approach is still poorly applied [21]. Recently, Lorusso et al. [27] successfully relied on the use of two primer pairs amplifying *COI* (targeting all the eukaryotes) and 12S rRNA genes (primarily targeting teleost fishes) to increase the confidence of species identification in processed seafood products. Dual-gene metabarcoding combining two primers can enhance the identification of species across a variety of complex food products, which is an area that has not been sufficiently addressed in prior research.

Based on these premises, a double-gene (16S rRNA and 12S rRNA) metabarcoding approach, relying on the inclusion of two different primer pairs (one, 12S rRNA, focused on fishes, the other, 16S rRNA, developed for metazoans and therefore broadly encompassing all animal species), was used to provide a complete overview of the composition of shrimp surimi-based products purchased from Chinese e-commerce platforms. Possible mislabeling cases were detected by comparing the molecular results with the information reported on the product labels; in addition, the environmental impact of the shrimp surimi-based products was evaluated by checking the status of detected shrimp and fish species on the IUCN Red List.

## 2. Materials and Methods

### 2.1. Sampling

**Figure 1:**
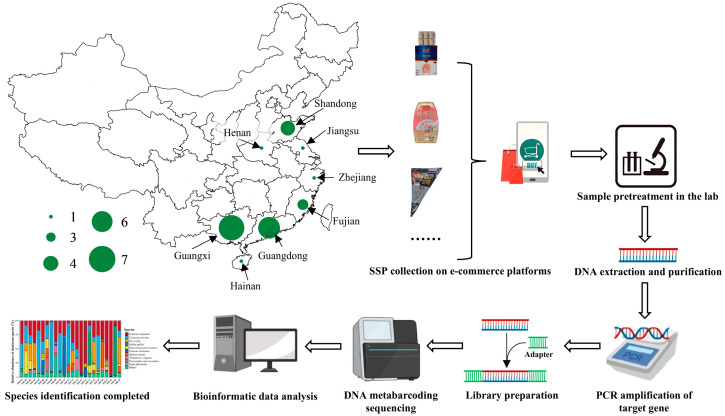
Shrimp surimi-based product (SSP) origin distribution (provinces), sample collection, and species identification and analysis processes. The experimental workflow illustrated in Figure 1 was created online at Biorender.com, accessed on 20 November 2024.

The sample collection, sequencing, and analysis workflow for this study are illustrated in Figure 1. Specifically, a total of 72 shrimp surimi-based products (SSPs) were purchased from five different Chinese e-commerce platforms from March to July 2024. These 72 products included triplicates of the same product type, so that 24 different SSPs were collected and analyzed (see Section 2.2). The SSPs were chosen by non-probability convenience sampling to include products from different brands. For each different SSP (*n* = 24), the production area (city or county, province), ingredient list, and price per kilogram (converted to USD) were translated into English and recorded (Table 1). The commercial names (in Chinese) of the shrimp species reported in the ingredient lists were queried against the SeaLifeBase (https://www.sealifebase.ca/search.php?lang=scChinese; accessed on 18 November 2024) and the China Animal Scientific Database (http://www.zoology.csdb.cn; accessed on 18 November 2024) to obtain the species scientific name, when possible.

**Table 1:** Shrimp surimi-based product (SSP) analyzed in this study, with corresponding production area, list of ingredients, and price.

Sample ID	Production Area (City or County, Province)	List of Ingredients	Declared Shrimp Species/Chinese Commercial Name	Other Declared Meat Species	Price (USD/kg)
SSP-01	Weifang City, Shandong Province	Shrimp (N.W. ≥ 95%), fish surimi, vegetable oil, starch, water, egg white, chicken, trehalose, salt, sodium glutamate, sugar, chicken essence seasoning, compound phosphate	—	—	23.98
SSP-02	Chengmai County, Hainan Province	Oriental river prawn (N.W. ≥ 65%), fish surimi, potato starch, egg white, salt, sugar, food additive	*M. nipponense*/青虾	—	13.07
SSP-03	Zhanjiang City, Guangdong Province	Shrimp (N.W. ≥ 95%), starch, salt, sodium glutamate, food additive	—	—	18.43
SSP-04	Dongxing City, Guangxi Zhuang Autonomous Region	Shrimp (N.W. ≥ 80%), cuttlefish (N.W. ≥ 10%), pig fat, fish surimi, water, salt, sugar, starch, sesame oil, spices, acetate starch, tapioca starch, compound phosphate, food seasoning	—	—	24.07
SSP-05	Anyang City, Henan Province	Oriental river prawn, fish surimi, starch, salt, egg white, sugar, sodium glutamate, vegetable oil, compound phosphate	*M. nipponense*/青虾	—	12.74
SSP-06	Zhangzhou City, Fujian Province	Shrimp (giant tiger prawn, Pacific white shrimp, N.W. ≥ 95%), starch, water, egg white, vegetable oil, salt, sugar, sodium glutamate, chicken essence seasoning, food additives	*P. monodon*/黑虎虾, *P. vannamei*/南美白对虾	—	40.39
SSP-07	Beihai City, Guangxi Zhuang Autonomous Region	Shrimp (N.W. ≥ 80%), fish surimi, starch, egg white, vegetable oil, trehalose, salt, sodium glutamate, compound phosphate	—	—	19.26
SSP-08	Zhangzhou City, Fujian Province	Shrimp, frozen fish surimi, drinking water, starch, trehalose, vegetable oil, salt, sodium glutamate, chicken powder seasoning, yeast extract, food additives	—	—	19.35
SSP-09	Rizhao City, Shandong Province	Shrimp, fish surimi (shrimp + fish surimi N.W. ≥ 95%), food additives, trehalose, water, vegetable oil, protein powder, salt, sodium glutamate, edible flavor	—	—	18.43
SSP-10	Zhangzhou City, Fujian Province	Shrimp, frozen fish surimi, starch, water, vegetable oil, egg white, salt, sodium glutamate, chicken powder seasoning, yeast extract, food additives	—	—	23.89
SSP-11	Beihai City, Guangxi Zhuang Autonomous Region	Giant tiger prawn (N.W. ≥ 95%), starch, egg white, vegetable oil, salt, water, sugar, chicken powder seasoning	*P. monodon*/黑虎虾	—	27.69
SSP-12	Shantou City, Guangdong Province	Shrimp (N.W. ≥ 85%), fish surimi, drinking water, starch, trehalose, sugar, compound acidity regulator, salt, sodium glutamate, egg white powder, frozen egg white, sodium tripolyphosphate, sodium hexametaphosphate, sodium pyrophosphate	—	—	25.83
SSP-13	Zhanjiang City, Guangdong Province	Oriental river prawn (N.W. ≥ 95%), water, egg white, pork fat, chicken powder seasoning, salt, trehalose, sodium citrate, sodium D-isoascorbate, moisture retainer, acetate starch, hydroxypropyl distarch phosphate	*M. nipponense*/青虾	—	24.35
SSP-14	Taizhou City, Jiangsu Province	Shrimp, fish surimi, edible starch, sugar, salt, sodium glutamate, egg protein, food additives	—	—	23.03
SSP-15	Beihai City, Guangxi Zhuang Autonomous Region	Shrimp (N.W. ≥ 90%), acetate starch, egg white, sugar, trehalose, salt, vegetable oil, sodium glutamate, compound water retention agent	—	—	34.17
SSP-16	Zhanjiang City, Guangdong Province	Giant tiger prawn (N.W. ≥ 95%), water, protein liquid, pork fat, chicken powder seasoning, salt, trehalose, sodium citrate, sodium D-isoascorbate, moisture retainer, acetate starch, hydroxypropyl distarch phosphate	*P. monodon*/黑虎虾	—	27.91
SSP-17	Beihai City, Guangxi Zhuang Autonomous Region	Oriental river prawn, fish surimi, squid, starch, egg white, salt, sugar, monosodium glutamate, vegetable oil, food additives	*M. nipponense*/青虾	—	18.43
SSP-18	Beihai City, Guangxi Zhuang Autonomous Region	Shrimp, pork, starch, water, egg white liquid, sugar, salt, chicken powder seasoning, food additives	—	—	22.57
SSP-19	Beihai City, Guangxi Zhuang Autonomous Region	Shrimp (N.W. ≥ 95%), fish surimi, potato starch, salt, frozen egg white, white sugar, chicken powder seasoning, compound water retaining agent	—	—	30.83
SSP-20	Shantou City, Guangdong Province	Shrimp, fish surimi, edible starch, egg white, trehalose, salt, sugar, sodium glutamate, vegetable oil, compound water retention agent	—	—	24.91
SSP-21	Weihai City, Shandong Province	Shrimp (N.W. ≥ 95%), fish surimi, water, starch, egg white liquid, sugar, salt, sodium glutamate, chicken powder seasoning, chicken broth seasoning, food additives	—	—	22.22
SSP-22	Jiaxing City, Zhejiang Province	Shrimp (N.W. ≥ 80%), frozen egg white, starch, vegetable oil, flying fish roe (N.W. ≥ 2%), salt, sugar, sodium glutamate, egg white powder, trehalose, compound moisture retainer, curdlan, edible flavors and spices	—	Exocoetidae	32.81
SSP-23	Weihai City, Shandong Province	Oriental river prawn (N.W. ≥ 95%), egg white, salt, peanut oil, starch, sodium glutamate	*M. nipponense*/青虾	—	13.70
SSP-24	Zhanjiang City, Guangdong Province	Oriental river prawn, water, water chestnuts, egg white liquid, pig fat, chicken powder seasoning, salt, trehalose, acetate starch, hydroxypropyl distarch phosphate, sodium citrate, sodium tripolyphosphate, sodium pyrophosphate	*M. nipponense*/青虾	—	11.94

N.W. means net weight.

### 2.2. DNA Extraction and PCR Amplification

From each of the 72 SSPs, a representative sample was produced by mixing the three identical units (for a total of 24 SSP samples). For each sample, approximately 25 mg of the mixture was transferred into sterile centrifuge tubes for subsequent total DNA extraction.

Total DNA was extracted from the 24 SSP samples using an E.Z.N.A.^®^ Water DNA Kit (Omega Bio-tek, Norcross, GA, USA) according to the manufacturer’s protocols. The target regions from the 16S rRNA (115 bp) and 12S rRNA (208 bp) genes were amplified by PCR using primers: 16s_Metazoa_fwd (5′-AGTTACYYTAGGGATAACAGCG-3′)/16s_Metazoa_rev (5′-CCGGTCTGAACTCAGATCAYGT-3′) [28] and V12S-U-F (5′-GTGCCAGCNRCCGCGGTYANAC-3′)/V12S-U-R (5′-ATAGTRGGGTATCTAATCCYAGT-3′) [29], respectively. PCR was performed in triplicate using 20 μL mixture containing 4 μL of 5 × FastPfu Buffer, 2 μL of 2.5 mM dNTPs, 0.8 μL of each primer (5 μM), 0.4 μL of FastPfu Polymerase, and 10 ng of template DNA. The following PCR program, performed identically for both primer pairs (16S rRNA and 12S rRNA), was used: 95 °C for 5 min; followed by 40 cycles at 95 °C for 30 s, 55 °C for 30 s, and 72 °C for 45 s; and a final extension at 72 °C for 10 min. The three PCR products of the same sample were mixed and verified using 2% agarose gel electrophoresis. Amplicons were extracted from 2% agarose gels and purified using an AxyPrep DNA Gel Extraction Kit (Axygen Biosciences, Union City, CA, USA) according to the manufacturer’s instructions. Finally, the purified DNA was quantified using a QuantiFluor™-ST fluorescent quantitation system (Promega, Madison, WI, USA).

### 2.3. Library Preparation and Sequencing

Library preparation and the sequencing of amplicons were performed by an external company (Shanghai BIOZERON Biotech. Co., Ltd., Shanghai, China). Specifically, purified PCR products were quantified using the Qubit^®^ 3.0 system (Life Invitrogen, Waltham, MA, USA). Subsequently, library preparation was conducted using a NEXTFLEX Rapid DNA-Seq Kit (Bioo Scientific, Austin, TX, USA). Then, the amplicon libraries were paired-end sequenced (2 × 150) on a DNBSEQ-G99 NGS platform (MGI, Shenzhen, Guangdong, China) according to the standard protocols.

### 2.4. Processing of Sequencing Data and Sequence Taxonomic Assignment

Raw fastq files were first demultiplexed using in-house perl scripts according to the barcode sequence information for each sample with the following criteria: (i) the 150 bp reads were truncated at any site receiving an average quality score < 20 over a 10 bp sliding window, discarding the truncated reads that were shorter than 50 bp; (ii) only reads that overlapped by more than 10 bp were assembled. Reads that could not be assembled were discarded. Reads were then processed to generate amplicon sequence variants (ASVs) using DADA2 in QIIME2 [30]. Subsequently, taxonomic analysis was conducted on the ASV representative sequences, and the results were normalized. Representative sequences for each ASV were taxonomically assigned using the blastn comparison method with a default e-value of 1 × 10^−10^ against the MitoFish database (Version 4.05, http://mitofish.aori.u-tokyo.ac.jp/download; accessed on 2 November 2024) for 12S rRNA and the Genbank database (Version 263.0, https://ftp.ncbi.nlm.nih.gov/blast/db/FASTA; accessed on 2 November 2024) for 16S rRNA.

For each sample, the number of sequences assigned to shrimp and fish was counted, resulting in a species annotation abundance table. The number of shrimp and fish detected at the species level in the samples was calculated based on the annotation results. Furthermore, the sequence counts for each sample were normalized, and bubble charts and bar graphs representing the biological composition of the samples were created using OmicStudio tools (Version 3.6; https://www.omicstudio.cn/tool?order=complex; accessed on 15 November 2024). The results highlighted the top 30 and 10 species in abundance, with the remaining species categorized as “Others”. Sankey diagrams illustrating the combinations of shrimp and fish species within the samples were also created using OmicStudio tools (Version 3.6).

### 2.5. Mislabeling Assessment and Evaluation of Environmental Impact

Considering that most of the products reported on the labels generic commercial designation (e.g., “shrimp”, or “fish”) that could not be assigned to a single specific species, mislabeling was evaluated based on the correspondence between the ingredients (only those of animal origin) reported on the product label and the molecular results. To perform this, the sequences assigned to shrimp and fish species were all included in the macro-categories “shrimp” and “fish”, respectively, and their presence was checked against the ingredient lists. Therefore, we opted to use 16S rRNA sequencing results, which can identify a wide range of animal-derived ingredients, to carry out this step. When the specific commercial designation of the shrimp was instead declared on the label, a comparison with the molecularly detected species was also performed.

As a supplement, the 12S rRNA sequencing results, aimed at fish species identification, were employed to enhance the detail of fish species used during SSP production.

Finally, referencing the methods of Detcharoen et al. [31] with some modifications, the percentages of shrimp and fish species in the SSPs were plotted on a bar graph according to each species’ IUCN Red List status, vulnerability to fishing, resilience, and price categories to holistically assess the ecological sustainability of production. This information was specifically sourced from SeaLifeBase (https://www.sealifebase.ca/search.php, last accessed on 24 November 2024) and the CABI Compendium (https://www.cabidigitallibrary.org/journal/cabicompendium, last accessed on 24 November 2024). Additionally, the IUCN Red List status for some species was retrieved from the IUCN Red List website (https://www.iucnredlist.org/, last accessed on 24 November 2024).

## 3. Results and Discussion

### 3.1. Sampling and SSP Declared Composition

In this study, 72 SSPs (identical in triplicates, for a total of 24 different SSPs samples) were purchased through five Chinese e-commerce platforms (Taobao, JD.com, Pinduoduo, Meituan, and Douyin). As shown in Figure 1, the origins of the 24 SSPs were primarily concentrated in Guangxi (7 SSPs; 29.17%) and Guangdong (6 SSPs; 25.00%), followed by Shandong (4 SSPs; 16.67%) and Fujian (3 SSPs; 12.50%), with Hainan, Zhejiang, Jiangsu, and Henan each accounting for 1 SSP (4.17%). Similar to that of other surimi-based products, SSP production in China is predominantly concentrated in the southeastern coastal provinces (such as Guangdong, Guangxi, and Fujian), regions that have easy access to raw materials for SSPs and a well-established seafood processing industry [32,33].

As shown in Figure 1, shrimp was mixed with other ingredients in the form of incomplete granules and pastes before being packaged. Only three shrimp trade names (Oriental river prawn, Pacific white shrimp, and giant tiger prawn) were reported on the ingredient lists of nine SSPs, with the rest using a generic trade name (“shrimp”) not associated with any taxonomic rank (see Table 1). By consulting the SeaLifeBase and China Animal Scientific Database, these three names could be linked to *M. nipponense*, *P. vannamei*, and *P. monodon*, respectively. These species actually correspond to some of the most heavily fished or farmed shrimp species according to the China Fishery Statistical Yearbook [3]. Additionally, apart from SSP-09, which only declared a combined percentage of ≥95% for shrimp and fish surimi, the ingredient lists of 15 SSPs declared a percentage of shrimp (or shrimp species) ranging from 65 to 95%. Notably, nine SSPs declared the presence of more than 95% of shrimp.

Additionally, ingredients of animal origin different from shrimps such as fish surimi (or fish roe), pork, chicken, and cuttlefish were also reported on the ingredient lists (see Table 1). Among the analyzed products, SSP-4 declared a presence of cuttlefish (cephalopods) of ≥10%, while SSP-22 declared ≥10% of flying fish roe. Notably, fish surimi was the major component in 14 SSPs, ranking fourth to second in the ingredient list, although detailed information about the type and proportion of fish used in surimi was not disclosed. This is in line with the Chinese National Standard for Food Safety—General Standard for the Labeling of Prepackaged Foods [34], which does not require the listing of the original ingredients of composite ingredients (here, referring to surimi) when they make up less than 25% of the product.

The cost of raw materials for the food industry is a significant component of the final price, which in turn directly influences consumer choices [35]. Consumers naturally desire good quality at low prices; however, producers often rely on the use of other species, often cheaper, to reduce production costs and increase profits [36]. Such replacements are particularly easily executed in highly processed fish products like surimi due to their mixed or composite nature and the difficulty for consumers in identifying specific ingredients. Zhang et al. [7] reported that cases of mislabeling involving the presence of other species among the ingredients in amounts higher than the primary ones (e.g., amount of pork higher than the amount of fish) may occur in molecularly analyzed surimi-based products. Similarly, other studies have highlighted the replacement of higher-value seafood with lower-cost alternatives, such as the substitution of prawn roe with capelin roe [36] or high-value fish species with farmed species like freshwater fish (Nile tilapia, Roho labeo, and silver carp) in processed products [31]. These practices not only mislead consumers but also pose ethical and dietary concerns, especially for individuals with religious dietary restrictions or allergies [9]. Furthermore, the prevalence of such practices reflects systemic issues in seafood supply chains, suggesting the need for more stringent regulatory oversight and advanced molecular techniques, such as metabarcoding, to improve transparency and sustainability in the production of processed seafood.

In some SSPs, shrimp was the main ingredient, with some samples declaring shrimp content between 65% and 95%. To analyze the relationship between shrimp content and SSP pricing, all SSPs were categorized into four groups: <80%, 80–90%, ≥90%, and those without declared shrimp content. These groups comprised 1, 4, 10, and 9 SSPs, respectively. Regarding price, the price range of all SSPs varied significantly, spanning from USD 11.94 to USD 40.39 per kilogram, with an average price of USD 23.08 per kilogram (Table 1). Regarding the species of shrimp used, *P. vannamei*, *P. monodon*, and *M. nipponense* exhibited notable differences in market prices due to variations in production costs, consumer preferences, and farming practices [37,38]. Among these, *P. monodon* was the most expensive, followed by *M. nipponense* and *P. vannamei*. Consequently, a higher proportion of *P. monodon* in SSPs likely led to an increase in production costs and higher prices. Kroetz et al. [39] reported that the giant tiger prawn (*P. monodon*) is one of the top two prawns by volume in the US market, indicating its significant market impact globally. The most expensive SSP, SSP-06, priced at USD 40.39 per kilogram, was the only product declaring the use of both *P. vannamei* and *P. monodon*. Other SSPs like SSP-11 and SSP-16, which declared over 95% shrimp content of *P. monodon*, had an average price of USD 27.80 per kilogram. SSPs declaring the inclusion of *M. nipponense* or those labeled with the generic term “shrimp” had average prices of USD 15.71 and USD 24.25 per kilogram, respectively. In terms of declared shrimp content, only SSP-02 had a shrimp content below 80%, priced at USD 13.07 per kilogram. Four SSPs had shrimp content ranging between 80% and 90%, with an average price of USD 25.49 ± 5.61 per kilogram. Most products (10 SSPs) declared a shrimp content of 90% or higher, with an average price of USD 26.37 ± 7.69 per kilogram. For the nine SSPs that did not declare shrimp content, the average price was USD 19.48 ± 4.68 per kilogram. Overall, the results of the price analysis indicate that SSPs with a higher percentage of shrimp correlate with higher product prices.

### 3.2. Molecular Analysis and Taxonomic Assignment

In this study, we decide to apply a double-gene approach. Indeed, with respect to the analysis of surimi-based products using DNA barcoding, Ooi et al. [15] considered that the dual-marker approach is more effective than the single-marker technique. Günther et al. [40] were able to identify multiple species in surimi products by combining full- and mini-DNA barcoding as a result of different primer affinities. Similarly, Giusti et al. [21] applied DNA barcoding with COI and 16S rRNA genes to accurately assess the species composition in fish burgers. Zhang et al. [7] also relied on both COI and cytb genes. The advantages of using a double-gene approach were also reported for metabarcoding analysis [27]. Regarding DNA barcoding, it is quite intuitive that using multiple genes can enhance analytical performance in multi-species products (given that this technique can only detect one species at a time), and the simultaneous use of two genes in metabarcoding is more innovative. In fact, considering that the technique itself can detect multiple species simultaneously (even using a single gene), the advantage of the multigenic approach lies in the fact that taxonomic coverage can be increased by using primer pairs that are specific for certain taxonomic ranks, resulting in a complementary action.

In this study, we employed double-gene primer pairs: one targeting fish species (12S rRNA) and the other broadly covering all animal species (16S rRNA) [28,29]. The sequencing results from the dual primers provided a more comprehensive assessment of the species composition potentially incorporated into SSPs. Specifically, the 16S rRNA sequencing results, potentially offering full coverage of all species declared in SSPs, were used to evaluate the labeling error rate of these products. Additionally, the 12S rRNA sequencing results served as a secondary verification for fish species incorporated into SSPs and facilitated the assessment of the ecological risks and production sustainability associated with the fish species used in SSP production. As indicated in Table S1, the amplification and NGS sequencing of both 16S rRNA and 12S rRNA target regions were successfully completed for all 24 analyzed samples, with an average number of valid reads per sample of 124,103 ± 10,213 for 16S rRNA and 128,152 ± 7992 for 12S rRNA. After bioinformatic analysis using the DADA2 R package, the percentage of retained reads for 16S rRNA was higher than 98.56%. For 12S rRNA, the percentage of retained reads exceeded 50% (ranging from 50.39% to 99.92%) in all samples except for SSP-04 (6.34%), SSP-11 (36.35%), SSP-16 (3.72%), SSP-22 (18.53%), and SSP-23 (0.73%).

In the research conducted on food authentication using metabarcoding, there is no consensus on a DNA threshold considered indicative of contamination [7,9]. Some researchers use specific percentages of relative species abundance, such as 0.01%, 0.1%, 1%, or 2%, as threshold values to filter results, while others filter data based on the rank order of species relative abundance [31,41,42,43]. In this study, based on the species annotations obtained from 16S and 12S rRNA sequencing, a 1% threshold was used for filtering. The species annotation results were presented in terms of the relative abundance of the TOP10 and TOP30 species to better cover the species mixed into the SSPs.

**Figure 2:**
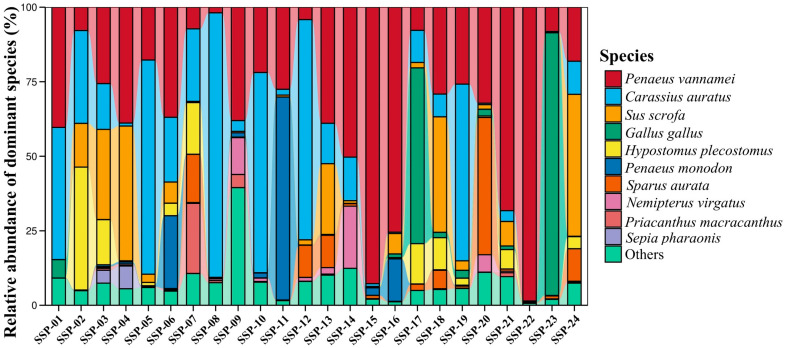
Barplot showing the abundance of species detected by the 16S rRNA primer pairs for each sample. The results of barplots only show the top 10 species in abundance.

The species annotation results from 16S rRNA sequencing indicated a rich diversity of species within SSPs, encompassing three phyla (Arthropoda, Chordata, and Mollusca) and six classes, which included Actinopterygii, Aves, Cephalopoda, Hexanauplia, Malacostraca, and Mammalia. Within these classes, 18 orders, 21 families, 29 genera, and 29 species were identified. With regard to species, *Penaeus* spp. (primarily *P. vannamei* and *P. monodon*) were widely present across 24 samples, exceeding 50% of reads in 7 samples, as shown in Table S2 and Figure 2. However, as shown in Figure 2, *M. nipponense*, which was the main ingredient (declared addition amount was in the range of ≥65–96%) on ingredient lists from some SSPs, was not found. Moreover, pork (*Sus scrofa*) and chicken (*Gallus gallus*) were also broadly identified across samples, with *S. scrofa* showing a high abundance in SSP-03 (30.24%), SSP-04 (45.19%), and SSP-24 (47.60%) and *G. gallus* in SSP-17 (58.94%) and SSP-23 (88.11%).

Species substitution in SSPs raises significant issues, including consumer deception due to mislabeling, ethical concerns for those with dietary restrictions, and potential health risks from undeclared allergens like pork and chicken. This study revealed frequent discrepancies between declared and identified ingredients, with primary components such as shrimp replaced by cheaper alternatives like pork and chicken. Such practices undermine trust in food labeling, compromise product quality, and highlight the need for stricter regulatory oversight.

**Figure 3:**
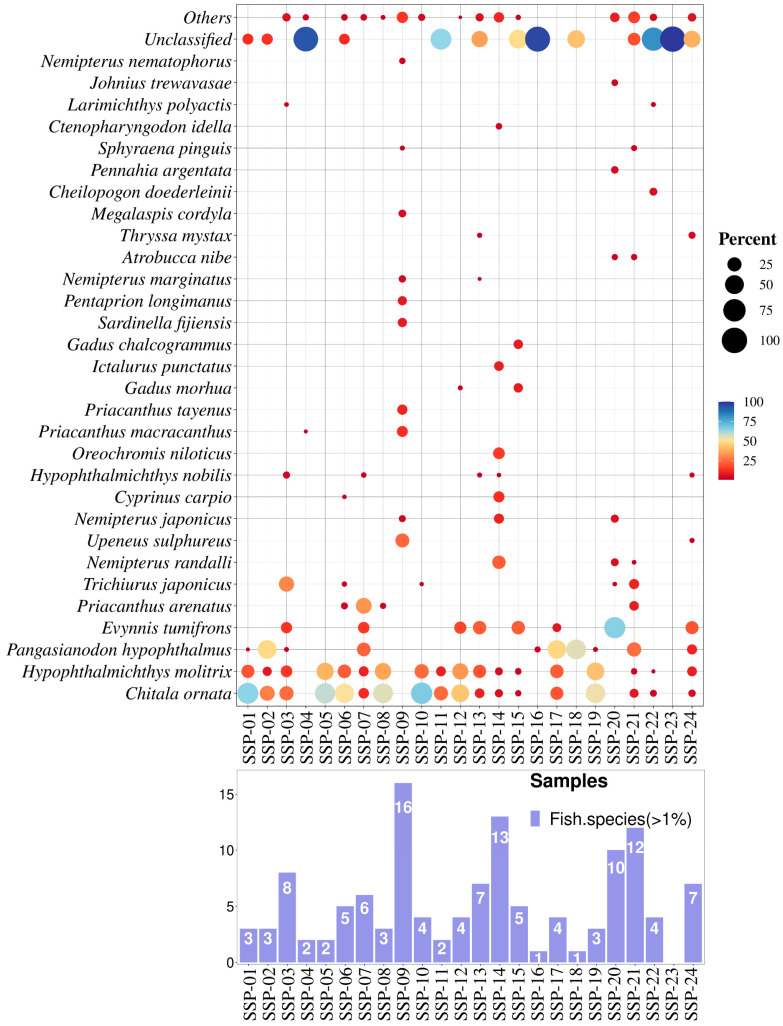
Analyses of the abundance of fish species detected by the 12S rRNA primer pairs in 24 SSPs. Percent abundance of the top 30 fish species in each SSP. Only fish species representing more than 1% of each sample are presented. Number of fish species used in each SSP. Only fish species representing more than 1% were considered.

With respect to 12S rRNA, we focused on the top 30 most abundant fish species in the SSP samples, spanning both freshwater and marine species (Figure 3). In the analyzed SSPs, the average number of fish species detected was about 5.2, with a range from 0 to 16 species per sample (relative abundance more than 1%). Specifically, the lowest number of species was detected in sample SSP-23, while the highest diversity was found in sample SSP-09. Zhang et al. [7] also found that surimi products purchased from Chinese e-commerce platforms contained from 2 to 11 different species per sample, with an average of 5.8 species, which is consistent with our findings. In recent years, the production of raw or frozen surimi in China has increasingly relied on freshwater fish species due to their high yield and low cost of production [44]. In this study, freshwater species identified in the SSPs using 12S rRNA included members of the Cyprinidae family such as silver carp (*Hypophthalmichthys molitrix*), common carp (*Cyprinus carpio*), bighead carp (*Hypophthalmichthys nobilis*), and grass carp (*Ctenopharyngodon idella*), as well as Nile tilapia (*Oreochromis niloticus*) and channel catfish (*Ictalurus punctatus*), all of which are extensively farmed and are considered candidates for surimi production [44]. Notably, clown featherback (*Chitala ornata*) was identified with an abundance exceeding 50% in six SSPs and over 25% in ten SSPs. This species is rarely found on Chinese dining tables but is popular in Southeast Asia, suggesting that Chinese producers may import this fish or its frozen surimi for the production of shrimp surimi [45]. Similarly, striped catfish (*P. hypophthalmus*), another economically significant fish species from Southeast Asia, was found in four SSPs with abundances over 25% [46]. This species is often confused with *Pangasius bocourti* and collectively marketed as “basa fish” to produce fish fillets and surimi [47,48]. It has high-quality, boneless, white flesh and is used by many as a cheaper alternative to cod [48]. In samples SSP-02, SSP-17, and SSP-18, its relative abundance reached 48.78%, 48.13%, and 56.09% respectively. Additionally, two species of *Gadus* (*G. morhua* and *G. chalcogrammus*), four species of *Nemipterus* (including *N. randalli*, *N. japonicus*, *N. marginatus*, and *N. nematophorus*), and three species of *Priacanthus* (*P. arenatus*, *P. macracanthus*, and *P. tayenus*) were also identified in some of the SSPs we analyzed. Accordingly, species from these three genera are commonly found in the production of surimi-based foods [31,36,49]. However, as shown in Figure 3, the overall frequency of occurrence and abundance of their sequences in SSPs were relatively low compared to those of the top three species. Additionally, the large-head hairtail (*Trichiurus japonicus*), one of the most heavily fished species in China’s marine fisheries, was also found in this study [3,50]. Notably, it constituted significant proportions in SSP-03 and SSP-21, with relative abundance reaching 30.07% and 10.79%, respectively.

Based on our results, it is evident that SSPs produced in China may incorporate substantial amounts of fish meat or its derivatives, often derived from cheaper or less palatable fish species. The addition of shrimp provides an opportunity for these fish species to be utilized as food ingredients. In principle, this practice is not inherently problematic, as it creates opportunities to use or repurpose inexpensive and less desirable fish species for processed food production. However, the significant discrepancies between the declared and detected proportions of species in the SSPs highlight major labeling issues.

### 3.3. Mislabeling Assessment and Evaluation of Environmental Impact

#### 3.3.1. Mislabeling Assessment

**Figure 4:**
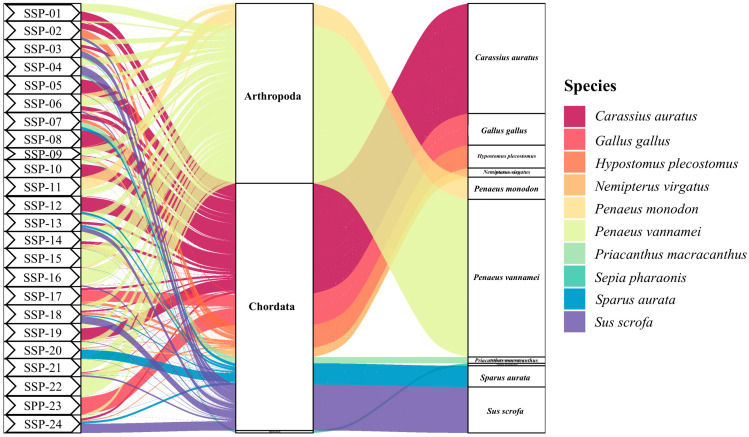
Sankey diagram showing the abundance of species detected by the 16S rRNA primer pairs and SSPs of labeled species. The results of Sankey diagram only show the top 10 species in abundance.

Using the 16S rRNA target, the declared animal-derived ingredients in the SSPs (shrimp, fish, chicken/egg white, pork, or cephalopod) were detected (sequence abundance ≥ 1%) in 45.8% of the samples (n = 11) (Table 2). In the remaining 54.2% (n = 13) of the samples, the declared presence of chicken, pork, or fish was not molecularly confirmed. Actually, in 5 of these 13 samples, a low number of sequences (below 1%) in these categories was first detected, but they were removed in the filtration step. Chicken was the category most involved in this occurrence, as four out of these five samples presented a below-threshold number of sequences. Therefore, considering the non-quantitative nature of this analysis, we cannot assume that those five samples were mislabeled in this sense. This implies that the presence of DNA from these species may have been linked to contamination during production. However, in our opinion, species identified above the threshold (≥1%) in this study remain reliable for evaluating mislabeling. Shrimp, the primary ingredient of the SSPs, was indeed detected in all the samples (100%), although it was not the most representative (in term of sequence abundance) in most cases. This highlights the robustness of the primers used, as their ability to detect shrimp sequences with high confidence also implies that the absence of sequences for other declared species can be confidently interpreted as their true absence (or presence only in trace amounts). This aspect further underscores the reliability of the analytical approach and warrants discussion regarding its implications for food authentication. Reiterating once again that this analysis is not quantitative but at most semi-quantitative, there are, however, cases where the high relative abundance of sequences from other categories declared on the label compared to that of shrimp may raise suspicions of intentional mislabeling. For instance, the shrimp sequence abundances were considerably lower than those of fish (SSP-02, SSP-05, SSP-07, SSP-08, SSP-10, SSP-12, SSP-19), pork (SSP-04, SSP-24), or chicken (SSP-17, SSP-23) in 11 samples (45.8%) (Table 2 and Figure 4). A “borderline” situation can be assumed for three samples (SSP-13, SSP-18, SSP-20), where the difference in the sequence abundances between shrimp and other categories was less pronounced, and based on the nature of the analysis, it cannot be stated that mislabeling occurred.

Regarding fish, their sequence abundances were particularly high, exceeding 50% in 9 out of 22 SSPs where fish was identified and surpassing 25% in 16 SSPs (Table 2). The declared addition of fish in SSPs was primarily in the form of fish surimi (14 SSPs) or fish roe (1 SSP). Although GB7718-2011 [34] regulations state that composite ingredients, such as fish surimi, do not require detailed composition disclosure when their content is below 25%, the significantly high sequence abundances of fish detected in this study are noteworthy.

In addition to this, undeclared categories (pork, fish, cephalopod, sea lice) were detected in most of the SSPs (66.7%; n = 16) (Table 2), thus exacerbating the situation in terms of mislabeling occurrence. Pork was especially found among undeclared ingredients, being present in 9 of these 16 samples, in some cases with considerably high sequence abundances (SSP-2; SSP-03) (Table 2). Research has shown that adding pork and pork fat as ingredients in surimi-based products can enhance the flavor and textural quality of the surimi [51]. However, adding undeclared pork not only constitutes food fraud but can also provoke disputes among vegetarians (such as pescatarians) and religious adherents [9,52]. Moreover, the identification of sea lice in SSP-07, typically parasitic on fish, raises concerns about the source and sanitary conditions of the raw materials used for SSP production [53]. The undeclared presence of cephalopods (mollusks), with a sequence abundance 6.11%, was also detected in one sample (SSP-03), exposing sensitive consumers to allergy risks [14].

Mollusk hypersensitivity is a common food allergy, with reactions ranging from mild oral symptoms to severe ones like anaphylactic shock in sensitive individuals. Tropomyosin (TM) was the first identified allergen, though other allergens have also been found [54]. According to European food regulations, the term “mollusks” must be clearly listed, as even trace amounts can pose a danger to allergic consumers (Regulation (EU) No 1169/2011). Moreover, under the guidance of the China National Food Safety Standard GB 7718-2011 [34], it is advisable to include a notice near the ingredient list about the use or possible inclusion of crustaceans, fish, and their products, which can provoke allergic reactions.

Only with respect to the nine SSP for which the species scientific name was available, it was observed that products claiming to contain Oriental river prawn (*M. nipponense*) (SSP-02, SSP-05, SSP-13, SSP-17, SSP-23, and SSP-24) were identified as being totally substituted with Pacific white shrimp (*P. vannamei*), as shown in Figure 4. This substitution may partially result from the misapplication of their common names. From an economic perspective, it is challenging to determine which species is more expensive. However, in terms of raw material availability, the total production of *P. vannamei* is nearly ten times that of *M. nipponense* [3]. For SSP-06, which declared the use of two shrimp species, *P. vannamei* and giant tiger prawn (*P. monodon*), both species were identified, consistent with the label list. Notably, in SSP-11 and SSP-16, which exclusively declared *P. monodon*, *P. vannamei* was also detected. In particular, SSP-16 showed a significantly higher abundance of *P. vannamei* than of *P. monodon* (Figure 4). Substituting *P. vannamei* for *P. monodon* could allow producers to reduce raw material costs and achieve illegal profit margins [37].

Based on the 16S rRNA results, 87.50% of the SSPs (21/24) were considered mislabeled (Table 2). Specifically, for products reporting shrimp species names on their labels, 88.89% (8/9) showed discrepancies with the species identified through 16S rRNA sequencing or involved substitutions with other shrimp species. This mislabeling rate is higher than the 61.9% mislabeling rate found in a molecular authentication study of surimi products sold on Chinese e-commerce platforms by Zhang et al. [7], highlighting how issues related to the surimi production chain are still concerning [55].

Our findings may also raise concerns about potential fish species substitution, although fish species declaration is not mandatory. For instance, as shown in Figure 4, the 16S rRNA sequencing results revealed the presence of goldfish (*Carassius auratus*) or suckermouth catfish (*H. plecostomus*) in all SSPs except SSP-15, SSP-16, SSP-20, and SSP-23. However, these species are not suitable for human consumption. The former is typically raised as an ornamental fish, while the latter is widely cultivated as an aquarium cleaner and rarely used as a food ingredient in surimi production [56,57]. The primary reason for these unreliable identifications could be attributed to the use of 16S rRNA amplicons, which are designed for broad animal identification but exhibit lower resolution for fish species [28]. This finding highlights the importance of employing a double-gene approach to achieve more accurate results when analyzing complex mixed food products through metabarcoding. Furthermore, it should be emphasized that the selected 12S rRNA amplicon is more informative for fish species identification. As shown in Figure 3, the 12S rRNA sequencing results revealed that SSP production mainly utilized *C. ornata*, *H. molitrix*, and fish species from the Cyprinidae family, which are extensively farmed in Asia. Substituting shrimp with these fish species in SSP production could significantly reduce costs. Instances of fish products replacing shrimp products for commercial purposes are not uncommon. For example, Ho et al. [36] reported that capelin roe (*Mallotus villosus*) was sold as prawn roe, highlighting similar mislabeling practices in seafood products.

#### 3.3.2. Fisheries Protection, Price, Resilience, and Vulnerability of Fish Species Used in SSPs

It is widely known that mislabeling may have negative impacts on fish populations and could therefore jeopardize the achievement of sustainable development goals [39]. The use of a wide range of species, sometimes vulnerable, is reported in surimi production [7,13,14], and this highlights the importance of accurately identifying species in such food products to better manage overexploited and/or endangered marine resources. Furthermore, the utilization of endangered marine resources in the seafood sector can have significant negative repercussions, including damage to company image and adverse publicity. For instance, the bycatch of endangered, threatened, and protected species in fishing operations has raised public concern and scrutiny toward companies involved in such practices [58]. Additionally, seafood production that contributes to biodiversity loss poses ethical and reputational challenges for companies sourcing from unsustainable fisheries [59]. Companies associated with unsustainable fishing practices risk reputational damage, underscoring the need for transparency and adherence to sustainability standards.

In this study, to evaluate the ecological sustainability and economic value of the main ingredients—shrimp and fish species—used in SSPs, we conducted a quantitative analysis based on the percentage of species identified from SSPs, using records from the IUCN Red List and either SeaLifeBase or FishBase. According to the IUCN Red List, both shrimp species found in the samples, *P. vannamei* and *P. monodon*, are classified as “Not Evaluated”. Additionally, they exhibit “High” resilience and “Low” fishing vulnerability, with a “Very High” price category (see Table S3). Moreover, in 2023, the aquaculture production of *P. vannamei* and *P. monodon* in China reached a staggering 2,238,390 tons (1,429,832 tons from marine aquaculture and 808,558 tons from freshwater aquaculture) and 128,420 tons (exclusively marine aquaculture), respectively [3]. These two shrimp species are primarily cultivated in marine environments, providing a convenient source of raw materials for the seafood processing industry in the coastal regions producing SSPs.

**Figure 5:**
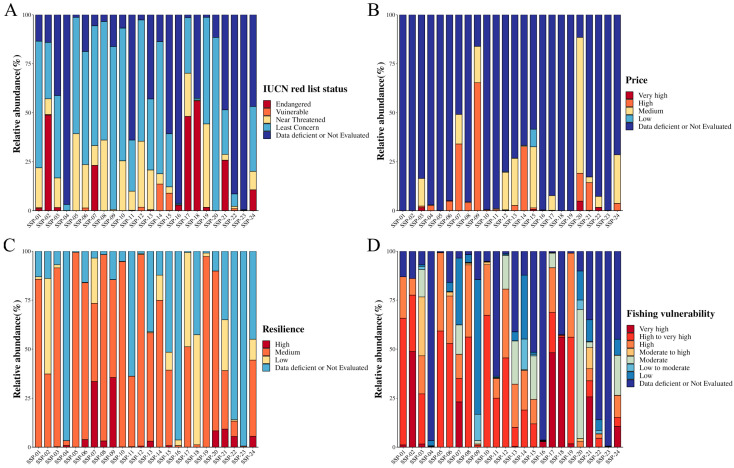
The percentages of the top 30 fish species in SSPs classified according to (**A**) the IUCN Red List status; (**B**) fishing vulnerability; (**C**) resilience; and (**D**) price.

In addition, to compare the sustainability and ecological risks of fish species used in SSP production, we selected the 12S rRNA sequencing results, as it primarily targets fish species, all of which can be compared using the same data platform, FishBase. Many fish species in the SSP samples were classified under the “Least Concern” and “Near Threatened” categories. They exhibited “Medium” and “High” resilience but “High” to “Very High” vulnerability to fishing, suggesting potential ecological risks in SSPs (see Figure 5). Notably, *P. hypophthalmus*, found in samples like SSP-1 (1.15%), SSP-02 (48.78%), SSP-03 (1.61%), SSP-07 (23.11%), SSP-16 (2.82%), SSP-17 (48.13%), SSP-18 (56.09%), SSP-19 (1.70%), SSP-21 (25.69%), and SSP-24 (10.66%), was the only species classified as “Endangered” (see Figure 5A, Table S3). This species is likely sourced mainly from aquaculture and processed into surimi-based products [31,46]. Other species like *C. carpio* (in SSP-06, and SSP-14, percentage > 1%) and *G. morhua* (in SSP-12, SSP-15, percentage > 1%) were classified as “Vulnerable”. There is limited information on the pricing of fish species mixed into SSPs, but some, like SSP-07, SSP-09, SSP-13, SSP-14, SSP-20, and SSP-24, used fish priced at “Low” to “High” levels to substitute for shrimp priced at “Very High” levels (see Figure 5B, Table S3). Species like *E. tumifrons*, *P. arenatus*, *N. randalli*, and *Upeneus sulphureus* were added to some SSPs in amounts exceeding 20%. Due to the high shrimp meat content in SSP-22, the addition of fish surimi had a minimal impact on the cost of production. Moreover, in eight samples (SSP-02, SSP-07, SSP-17, SSP-18, and SSP-21), more than 20% of the fish added were of the “Low” resilience category, such as *P. hypophthalmus*, *I. punctatus*, *C. idella*, and *G. chalcogrammus* (see Figure 5C; Table S3). According to FAO data, the global catch of *G. chalcogrammus* plummeted from ~7 mt in 1986 to ~3 mt in 2015 [60]. Furthermore, most fish species (such as *C. ornata*, *H. molitrix*, *P. hypophthalmus*, *C. carpio*, *H. nobilis*, *G. morhua*, *I. punctatus*, *G. chalcogrammus*, *A. nibe*, and *C. idella*) used in SSPs were classified as having “High” to “Very High” vulnerability to fishing (see Figure 5D; Table S3). Only in SSP-04, SSP-09, SSP-14, SSP-20, and SSP-22 were fish species with “Low” vulnerability to fishing such as *U. sulphureus*, *Cheilopogon doederleinii*, *P. macracanthus*, *N. japonicus*, *N. randalli*, *P. tayenus*, *P. arenatus*, *S. fijiensis*, *N. marginatus*, *T. mystax*, *J. trewavasae*, and *N. nematophorus* used. It is important to note that although fish species such as *C. ornata* and *Nemipterus* spp. are abundant in Southeast Asia and are important for consumption and surimi production, the latter has low vulnerability to fishing, indicating minimal impact from fishing and a short reproductive cycle [45,61]. Therefore, the fish meat or fish surimi mixed into SSPs, which does not require detailed disclosure of species in the food ingredient lists, poses a high ecological risk and potential for species substitution.

## 4. Conclusions

This study underscores the substantial potential of DNA metabarcoding technology for rapidly identifying multiple species within complex processed foods. The speed, cost-effectiveness, and versatility of this technology make it a powerful tool for monitoring and evaluating the intricate species composition in seafood products. Our analysis based on a double-gene approach was able to identify two principal shrimp species, *P. vannamei* and *P. monodon*, in 24 SSP samples. *P. vannamei*, the primary ingredient in Chinese e-commerce-sourced SSPs, was also found to be substituted for declared *P. monodon* in some SSPs. Additionally, the substitution with fish species such as *C. ornata*, *H. molitrix*, *P. hypophthalmus*, *Gadus* spp., *Nemipterus* spp., and *Priacanthus* spp. and with pork and chicken were observed. Overall, the mislabeling rate for SSPs sold on Chinese e-commerce platforms was as high as 87.50%, with only three SSPs being correctly labeled. Moreover, the shrimp and fish species used in the production of SSPs generally fall under the IUCN categories of least concern and near threatened, as well as having medium to high resilience. However, the use of fish species categorized as high to very high in fishing vulnerability indicates potential ecological risks in SSP production. These inconsistencies underscore the importance of employing advanced analytical methods, such as metabarcoding, to accurately verify the composition of SSPs and ensure transparency in product labeling. Addressing mislabeling is critical for maintaining consumer trust and upholding ethical practices in the seafood processing industry. Thus, the rise of DNA metabarcoding technology based on high-throughput sequencing is revolutionizing food safety monitoring and supply chain integrity [9,31,43]. DNA metabarcoding has proven to be an effective tool for ingredient authentication in processed seafood, capable of resolving species identities within complex mixtures [23].

## Figures and Tables

**Table 2:** Mislabeling assessment of SSPs based on 16S rRNA amplicon metabarcoding results.

Sample	Ingredients	16S rRNA	Other Not Declared (16S rRNA)	Declared Shrimp Species	16S rRNA	Other Not Declared (16S rRNA)	Mislabeling
SSP-01	Shrimp (≥95%)	Yes (45.41%)					No
	Fish	Yes (44.32%)					
	Egg white/chicken	Yes (6.16%)					
SSP-02	Shrimp (≥65%)	Yes (7.81%)	Pork (14.66%)	*M. nipponense*	No	*P. vannamei* (7.81%)	Yes
	Fish	Yes (72.40%)					
	Egg white/chicken	No *					
SSP-03	Shrimp (≥95%)	Yes (25.63%)	Cephalopod (6.11%)				Yes
			Fish (30.51%)				
			Pork (30.24%)				
SSP-04	Shrimp (≥80%)	Yes (38.88%)					No
	Cephalopod (≥10%)	Yes (10.85%)					
	Pork	Yes (45.20%)					
	Fish	Yes (1%)					
SSP-05	Shrimp ^[a]^	Yes (17.71%)	Pork (2.77%)	*M. nipponense*	No	*P. vannamei* (17.71%)	Yes
	Fish	Yes (72.97%)					
	Egg white/chicken	No					
SSP-06	Shrimp (≥95%)	Yes (61.40%)	Fish (25.92%)	*P. monodon*	Yes (36.93%)		Yes
	Egg white/chicken	No	Pork (7.09%)	*P. vannamei*	Yes (24.47%)		
SSP-07	Shrimp (≥80%)	Yes (7.24%)	Sea lice (4.01%)				Yes
	Fish	Yes (81.39%)					
	Egg white/chicken	No					
SSP-08	Shrimp	Yes (1.87%)					Yes
	Fish	Yes (88.73%)					
SSP-09	Shrimp (shrimp + fish ≥ 95%)	Yes (39.48%)					Yes
	Fish (shrimp + fish ≥ 95%)	Yes (53.76%)					
	Chicken	No					
SSP-10	Shrimp ^[a]^	Yes (23.61%)					Yes
	Fish	Yes (63.35%)					
	Egg white/chicken	No					
SSP-11	Shrimp (≥95%)	Yes (95.65%)	Fish (1.94%)	*P. monodon*	Yes (68.10%)	*P. vannamei* (27.55%)	Yes
	Egg white/chicken	No					
SSP-12	Shrimp (≥85%)	Yes (4.16%)	Pork (1.76%)				Yes
	Fish	Yes (85.95%)					
	Egg white/chicken	No *					
SSP-13	Shrimp (≥95%)	Yes (38.94%)	Fish (27.97%)	*M. nipponense*	No	*P. vannamei* (38.94%)	Yes
	Egg white/chicken	No *					
	Pork	Yes (23.74%)					
SSP-14	Shrimp ^[a]^	Yes (50.29%)					Yes
	Fish	Yes (43.25%)					
	Egg white/chicken	No					
SSP-15	Shrimp (≥90%)	Yes (95.34%)	Fish (1.08%)				Yes
	Egg white/chicken	No					
SSP-16	Shrimp (≥95%)	Yes (89.71%)		*P. monodon*	Yes (14.25%)	*P. vannamei* (75.46%)	Yes
	Egg white/chicken	Yes (1.24%)					
	Pork	Yes (6.85%)					
SSP-17	Shrimp ^[a]^	Yes (7.76%)	Pork (1.81%)	*M. nipponense*	No	*P. vannamei* (7.76%)	Yes
	Fish	Yes (26.41%)					
	Cephalopod	Yes (1.91%)					
	Egg white/chicken	Yes (58.96%)					
SSP-18	Shrimp ^[a]^	Yes (29.14%)	Fish (24.62%)				Yes
	Pork	Yes (38.71%)					
	Egg white/chicken	Yes (1.83%)					
SSP-19	Shrimp (≥95%)	Yes (25.82%)	Pork (3.18%)				Yes
	Fish	Yes (61.63.%)					
	Egg white/chicken	Yes (2.63%)					
SSP-20	Shrimp ^[a]^	Yes (32.20%)	Pork (1.55%)				Yes
	Fish	Yes (55.54%)					
	Egg white/chicken	Yes (2.24%)					
SSP-21	Shrimp (≥95%)	Yes (68.24%)	Pork (8.21%)				Yes
	Fish	Yes (15.40%)					
	Egg white/chicken	Yes (1.20%)					
SSP-22	Shrimp (≥80%)	Yes (98.57%)					No ^[b]^
	Egg white/chicken	No *					
	Fish (≥2%)	No *					
SSP-23	Shrimp (≥95%)	Yes (8.14%)	Fish (1.00%)	*M. nipponense*	No	*P. vannamei* (8.14%)	Yes
	Egg white/chicken	Yes (88.14%)					
	Pork	No *					
SSP-24	Shrimp (≥95%)	Yes (18.11%)	Fish (26.08%)	*M. nipponense*	No	*P. vannamei* (18.11%)	Yes
	Egg white/chicken	No *					
	Pork	Yes (47.63%)					

* Sequences abundance ≤ 1%. ^[a]^ Assumed to be below the 25% threshold. ^[b]^ The actual presence of chicken and fish in the product cannot be excluded.

## Data Availability

Data are contained within the article and Supplementary Materials.

## Supplementary Materials

The following supporting information can be downloaded at: https://www.mdpi.com/article/10.3390/genes16020144/s1, Table S1: Number of reads before and after filtering 16S rRNA sequencing sequences and number of reads before and after filtering 12S rRNA sequencing sequences; Table S2: Relative abundance of the top 30 species in SSPs detected by the 16S rRNA primer pairs and relative abundance of the top 30 species in SSPs detected by the 12S rRNA primer pairs; Table S3: Relative abundance of the top 10 species in SSPs detected by the 16S rRNA primer pairs, classified according to the IUCN Red List status, fishing vulnerability, resilience, and price, and the relative abundance of the top 30 fish species in SSPs detected by the 12S rRNA primer pairs, classified according to the IUCN red list status, fishing vulnerability, resilience, and price.
