# Supplementary material for: A Double-Gene Metabarcoding Approach for the Authentication of Shrimp Surimi-Based Products
Source: Genes (Basel). 2025 Jan 24;16(2):144. doi: 10.3390/genes16020144 (PMC11855189; doi:10.3390/genes16020144)
Supplement: Supplementary file 1 [file genes-16-00144-s001.zip › genes-3418239-supplementary.pdf]

Supplementary Materials

Table S1. Number of reads before and after filtering 16S rRNA sequencing sequences and number of reads before and after filtering 12S rRNA sequencing sequences.

**Samples**	**Sequences**	**Average Sequences Length (bp)**	**Reads Classification**	**Sequences Reads Belong to Unambigous Group**	**Assigned/%**	**Species Found（>2%）**
SSP-01	136513	114.63	134163	134159	100.00%	4
SSP-02	126831	114.92	125024	125020	100.00%	4
SSP-03	115192	114.74	112497	112492	100.00%	5
SSP-04	129268	114.6	126619	126612	99.99%	4
SSP-05	120286	114.82	118640	118636	100.00%	3
SSP-06	131920	114.36	129741	129371	99.71%	5
SSP-07	117427	114.93	115019	115019	100.00%	6
SSP-08	135607	114.98	134572	134561	99.99%	1
SSP-09	129000	114.62	126366	126345	99.98%	10
SSP-10	129817	114.77	128849	128825	99.98%	2
SSP-11	118976	113.89	117426	115737	98.56%	2
SSP-12	124383	114.96	123379	123379	100.00%	3
SSP-13	113939	114.62	112484	112482	100.00%	5
SSP-14	132935	114.5	131686	131685	100.00%	5
SSP-15	110927	114.03	110198	110110	99.92%	2
SSP-16	128634	114.1	127828	127653	99.86%	3
SSP-17	131393	115.55	130285	130283	100.00%	4
SSP-18	138378	114.73	137079	137072	99.99%	5
SSP-19	119434	114.76	118749	118749	100.00%	5
SSP-20	126151	114.71	123471	123471	100.00%	5
SSP-21	113810	114.37	112004	112004	100.00%	5
SSP-22	121671	114.02	120622	120622	100.00%	1
SSP-23	132722	115.82	130719	130719	100.00%	2
SSP-24	93257	114.84	91318	91316	100.00%	5
Samples	Sequences	Average sequences length (bp)	Reads classification	Sequences reads belong to unambigous group	Assigned/%	Species found（>2%）
SSP-01	134903	167.4	126475	109974	86.95%	4
SSP-02	133621	163.67	125872	108383	86.11%	3
SSP-03	116023	162.43	107646	107032	99.43%	5
SSP-04	131933	158.49	123047	7797	6.34%	5
SSP-05	127323	167.9	120400	119807	99.51%	5
SSP-06	139266	165.59	130913	114476	87.44%	5
SSP-07	115005	162.23	108408	108317	99.92%	1
SSP-08	136623	167.18	132942	132682	99.80%	2

SSP-09	124845	159.57	117172	116755	99.64%	5
SSP-10	129977	167.12	127021	125829	99.06%	4
SSP-11	135162	160.48	130667	47498	36.35%	5
SSP-12	113018	166.01	110370	110101	99.76%	4
SSP-13	134560	161.84	130081	84382	64.87%	3
SSP-14	136074	160.01	129436	128219	99.06%	5
SSP-15	114908	158.07	110742	55807	50.39%	6
SSP-16	133039	158.02	129208	4801	3.72%	1
SSP-17	130161	164.43	126670	126290	99.70%	10
SSP-18	127885	160.55	125054	71755	57.38%	2
SSP-19	122649	167.73	120211	119075	99.05%	2
SSP-20	139286	158.51	132843	131829	99.24%	5
SSP-21	120758	160.24	114920	93161	81.07%	5
SSP-22	121243	165.06	115431	21390	18.53%	2
SSP-23	126912	168.87	122583	889	0.73%	3
SSP-24	130463	160.69	125618	77448	61.65%	4

Table S2. The relative abundance of the top 30 species in SPPs detected by the 16S rRNA primer pairs.

Species Name	SSP-01	SSP-02	SSP-03	SSP-04	SSP-05	SSP-06	SSP-07	SSP-08	SSP-09	SSP-10	SSP-11	SSP-12	SSP-13	SSP-14	SSP-15	SSP-16	SSP-17	SSP-18	SSP-19	SSP-20	SSP-21	SSP-22	SSP-23	SSP-24
Ictalurus furcatus	0.00	0.03	0.02	0.00	0.00	0.01	0.02	0.01	0.00	0.07	0.00	0.23	0.12	2.33	0.02	0.00	0.01	0.01	0.00	0.00	0.02	0.00	0.00	0.00
Priacanthus tayenus	0.00	0.00	0.04	0.08	0.00	0.00	0.14	0.21	5.39	0.20	0.05	0.03	0.34	0.00	0.00	0.00	0.00	0.00	0.00	0.00	0.01	0.00	0.00	0.00
Unclassified	0.00	0.00	0.00	0.01	0.00	0.27	0.00	0.01	0.02	0.02	1.46	0.00	0.00	0.00	0.09	0.14	0.00	0.01	0.00	0.00	0.00	0.00	0.00	0.00
Caligus hamruri	0.00	0.00	0.09	0.00	0.00	0.06	4.01	0.06	0.00	0.01	0.00	0.00	0.00	0.00	0.00	0.00	0.00	0.00	0.02	0.01	0.25	0.00	0.01	0.00
Penaeus japonicus	1.44	0.01	0.00	0.00	0.00	0.01	0.01	0.00	0.01	0.01	0.06	0.00	0.01	0.00	0.01	0.01	0.00	0.00	0.00	0.00	0.01	0.00	0.00	0.00
Hypostomus plecostomus	0.01	41.21	15.14	0.36	1.10	4.18	17.33	0.00	0.01	0.00	0.00	0.03	0.12	0.01	0.01	0.44	13.53	10.77	2.38	0.51	6.53	0.03	0.15	4.05
Nemipterus mesoprion	0.00	0.00	0.06	0.11	0.01	0.02	0.21	0.13	4.83	0.15	0.04	0.08	1.40	0.06	0.04	0.01	0.02	0.34	0.02	0.01	0.00	0.00	0.00	0.53
Saurida tumbil	0.00	0.00	0.00	0.00	0.00	0.01	0.04	0.09	1.70	0.14	0.02	0.04	0.45	0.02	0.01	0.00	0.02	0.31	0.04	0.19	0.25	0.00	0.02	0.45
Priacanthus macracanthus	0.00	0.02	0.63	0.21	0.05	0.30	23.49	0.72	4.41	0.25	0.05	0.08	0.36	0.01	0.00	0.00	0.01	0.12	0.08	0.06	1.32	0.01	0.03	0.32
Nemipterus virgatus	0.00	0.01	0.27	0.49	0.04	0.20	0.29	0.61	12.34	1.18	0.11	1.27	2.18	20.83	0.21	0.01	0.01	0.13	0.06	5.83	0.52	0.04	0.13	0.31
Upeneus sulphureus	0.00	0.00	0.04	0.04	0.00	0.04	0.11	0.34	9.48	0.30	0.07	0.05	0.93	0.08	0.01	0.00	0.01	0.11	0.03	0.07	0.30	0.00	0.01	0.23
Nemipterus peronii	0.00	0.00	0.44	0.71	0.06	0.06	0.42	0.04	1.03	0.06	0.01	0.04	0.45	0.04	0.00	0.00	0.01	0.07	0.01	0.06	0.00	0.00	0.00	0.16
Gallus gallus	6.16	0.01	0.01	0.01	0.00	0.00	0.00	0.00	0.00	0.00	0.00	0.01	0.07	0.00	0.00	1.24	58.94	1.83	2.63	2.25	1.20	0.36	88.11	0.13
Cirrhitichthys aprinus	0.00	0.00	0.00	0.00	0.00	0.00	0.05	0.01	0.00	0.00	0.00	0.30	0.03	0.02	0.02	0.00	0.01	0.02	0.00	2.54	0.04	0.02	0.05	0.10
Pomadasys maculatus	0.00	0.00	0.01	0.00	0.00	0.05	0.02	0.11	0.02	0.32	0.00	0.39	0.24	4.38	0.04	0.00	0.02	0.02	0.04	1.11	0.66	0.01	0.05	0.06
Sinibotia robusta	0.71	0.44	0.20	0.02	0.45	0.33	0.57	0.70	4.80	0.72	0.07	0.51	0.49	0.17	0.01	0.00	0.05	0.06	0.63	0.01	0.09	0.00	0.00	0.06
Nemipterus japonicus	0.00	0.00	0.04	0.07	0.01	0.01	0.03	0.06	1.50	0.13	0.02	0.07	0.23	1.07	0.02	0.00	0.00	0.01	0.01	0.65	0.04	0.00	0.03	0.05
Trichiurus lepturus	0.00	0.01	0.02	0.00	0.01	0.36	0.01	0.36	0.01	0.48	0.01	0.03	0.00	0.02	0.00	0.01	0.00	0.00	0.19	0.90	3.90	0.02	0.20	0.03
Sander vitreus	0.00	0.00	0.00	0.00	0.00	0.00	0.00	0.16	2.26	0.11	0.01	0.03	0.17	0.02	0.00	0.00	0.00	0.01	0.01	0.04	0.18	0.00	0.00	0.03
Sus scrofa	0.02	14.66	30.24	45.19	2.77	7.07	0.41	0.05	0.45	0.04	0.64	1.76	23.72	0.87	0.41	6.84	1.81	38.69	3.18	1.55	8.20	0.06	0.34	47.60
Penaeus vannamei	40.32	7.81	25.61	38.86	17.71	36.82	7.25	1.87	37.98	21.91	27.14	4.16	38.93	50.27	92.66	75.35	7.76	29.12	25.81	32.17	68.20	98.56	8.13	18.10
Carassius auratus	44.27	31.15	15.35	1.00	71.81	21.64	24.33	88.68	3.61	67.12	1.91	73.82	13.54	14.61	0.94	0.44	10.78	7.63	59.24	0.49	3.63	0.09	0.10	11.10
Sparus aurata	0.00	0.01	0.34	0.00	0.02	0.01	16.16	0.16	0.20	0.03	0.05	10.82	10.83	0.87	1.08	0.13	2.09	6.21	0.70	46.01	0.74	0.18	1.00	10.91
Penaeus monodon	0.01	0.19	0.60	0.68	0.34	24.40	0.00	0.30	1.44	1.70	67.09	0.01	0.09	0.08	2.57	14.24	0.08	0.16	0.30	0.00	0.00	0.00	0.00	0.00
Sepia pharaonis	0.00	0.00	4.33	7.64	0.17	0.32	0.02	0.00	0.00	0.00	0.00	0.00	0.00	0.00	0.00	0.00	0.00	0.00	0.00	0.01	0.00	0.00	0.00	0.00
Centropomus ensiferus	0.00	0.00	0.01	0.02	0.00	0.00	0.00	0.05	2.44	0.05	0.02	0.00	0.19	0.00	0.00	0.00	0.00	0.00	0.00	0.00	0.00	0.00	0.00	0.00
Dosidicus gigas	0.00	0.00	0.00	0.00	0.00	0.00	0.00	0.00	0.00	0.00	0.00	0.00	0.00	0.00	0.00	0.13	1.92	0.05	0.09	0.00	0.00	0.00	0.00	0.00
Macrobrachium malcolmsonii	1.23	0.00	0.00	0.00	0.00	0.00	0.00	0.00	0.00	0.00	0.00	0.00	0.00	0.00	0.00	0.00	0.00	0.00	0.00	0.00	0.00	0.00	0.00	0.00
Sepia ramani	0.00	0.00	1.78	3.22	0.08	0.14	0.01	0.00	0.00	0.00	0.00	0.00	0.00	0.00	0.00	0.00	0.00	0.00	0.00	0.00	0.00	0.00	0.00	0.00
Mierspenaeopsis hardwickii	2.46	0.00	0.00	0.00	0.00	0.00	0.01	0.00	0.00	0.00	0.00	0.00	0.00	0.00	0.00	0.00	0.00	0.00	0.00	0.00	0.00	0.00	0.00	0.00
Others	3.37	4.44	4.73	1.28	5.37	3.69	5.06	5.27	6.07	5.00	1.17	6.24	5.11	4.24	1.85	1.01	2.92	4.32	4.53	5.53	3.91	0.62	1.64	5.78

The relative abundance of the top 30 species in SPPs detected by the 12S rRNA primer pairs.

Species Name	SSP-01	SSP-02	SSP-03	SSP-04	SSP-05	SSP-06	SSP-07	SSP-08	SSP-09	SSP-10	SSP-11	SSP-12	SSP-13	SSP-14	SSP-15	SSP-16	SSP-17	SSP-18	SSP-19	SSP-20	SSP-21	SSP-22	SSP-23	SSP-24
Chitala ornata	64.55	28.80	25.61	0.10	59.25	52.69	12.02	56.14	0.98	67.14	25.02	43.77	9.63	6.05	3.00	0.25	20.61	0.84	54.39	0.03	7.95	3.85	0.16	4.59
Hypophthalmichthys molitrix	20.24	8.02	15.08	0.07	39.24	22.12	10.09	36.01	0.57	25.38	9.72	33.67	20.25	5.23	3.26	0.23	22.02	0.36	42.57	0.03	2.95	1.15	0.10	9.43
Pangasianodon hypophthalmus	1.15	48.78	1.61	0.02	0.01	0.05	23.11	0.01	0.04	0.04	0.00	0.01	0.42	0.03	0.11	2.82	48.13	56.09	1.70	0.02	25.69	0.09	0.11	10.66
Evynnis tumifrons	0.00	0.00	14.01	0.08	0.00	0.00	14.99	0.09	0.02	0.00	0.31	17.16	21.97	0.46	22.14	0.26	7.42	0.00	0.03	65.77	0.16	0.10	0.01	20.27
Priacanthus arenatus	0.00	0.00	0.03	0.00	0.09	3.87	33.18	2.81	0.04	0.14	0.00	0.00	0.01	0.04	0.00	0.00	0.01	0.00	0.00	0.01	9.17	0.00	0.04	0.08
Trichiurus japonicus	0.00	0.00	30.07	0.16	0.09	2.02	0.01	0.87	0.01	1.30	0.01	0.00	0.01	0.00	0.00	0.00	0.01	0.00	0.00	1.41	10.79	0.00	0.06	0.07
Nemipterus randalli	0.00	0.00	0.00	0.00	0.01	0.32	0.03	0.26	0.03	0.03	0.00	0.07	0.00	22.10	0.10	0.01	0.00	0.01	0.00	5.39	1.48	0.01	0.01	0.09
Upeneus sulphureus	0.00	0.00	0.54	0.30	0.00	0.00	0.33	0.22	24.26	0.00	0.24	0.01	0.96	0.00	0.01	0.00	0.06	0.00	0.00	0.02	0.02	0.01	0.00	1.76
Nemipterus japonicus	0.00	0.00	0.11	0.53	0.02	0.49	0.20	0.29	4.02	0.07	0.03	0.11	0.21	10.47	0.27	0.02	0.05	0.00	0.00	5.90	0.28	0.00	0.00	0.14
Cyprinus carpio	0.48	0.28	0.02	0.00	0.05	1.34	0.02	0.02	0.01	0.02	0.24	0.15	0.01	13.46	0.10	0.00	0.01	0.01	0.00	0.00	0.06	0.99	0.01	0.00
Hypophthalmichthys nobilis	0.42	0.17	4.26	0.02	0.63	0.63	2.12	0.71	0.01	0.25	0.08	0.99	1.71	1.39	0.18	0.01	0.80	0.00	0.34	0.00	0.07	0.02	0.00	1.75
Oreochromis niloticus	0.00	0.00	0.01	0.00	0.00	0.00	0.02	0.00	0.00	0.01	0.00	0.07	0.02	15.55	0.11	0.00	0.07	0.01	0.00	0.00	0.01	0.01	0.00	0.00
Priacanthus macracanthus	0.00	0.00	0.02	1.14	0.00	0.00	0.00	0.09	13.02	0.00	0.08	0.02	0.31	0.00	0.00	0.00	0.04	0.00	0.00	0.00	0.01	0.01	0.00	0.65
Priacanthus tayenus	0.00	0.00	0.00	0.40	0.00	0.00	0.00	0.10	11.02	0.00	0.06	0.00	0.01	0.00	0.00	0.00	0.01	0.00	0.00	0.00	0.00	0.00	0.00	0.02
Gadus morhua	0.00	0.00	0.00	0.00	0.00	0.00	0.00	0.00	0.01	0.02	0.00	1.64	0.08	0.11	8.70	0.00	0.00	0.00	0.00	0.00	0.02	0.00	0.00	0.00
Ictalurus punctatus	0.00	0.00	0.00	0.00	0.00	0.00	0.00	0.00	0.00	0.00	0.00	0.05	0.00	9.50	0.06	0.00	0.00	0.00	0.00	0.00	0.00	0.01	0.00	0.00
Gadus chalcogrammus	0.00	0.00	0.00	0.00	0.00	0.00	0.00	0.00	0.00	0.00	0.00	0.35	0.06	0.05	8.95	0.00	0.00	0.00	0.00	0.00	0.00	0.00	0.00	0.00
Sardinella fijiensis	0.00	0.00	0.04	0.00	0.00	0.00	0.05	0.07	8.42	0.00	0.08	0.00	0.05	0.00	0.00	0.00	0.00	0.00	0.00	0.00	0.01	0.00	0.00	0.32
Pentaprion longimanus	0.00	0.00	0.00	0.25	0.00	0.00	0.00	0.09	8.19	0.00	0.06	0.00	0.00	0.00	0.00	0.00	0.00	0.00	0.00	0.00	0.01	0.00	0.00	0.00
Nemipterus marginatus	0.00	0.00	0.09	0.19	0.00	0.00	0.27	0.05	4.57	0.00	0.03	0.01	1.10	0.01	0.35	0.00	0.04	0.00	0.00	0.00	0.01	0.00	0.00	0.85
Atrobucca nibe	0.00	0.00	0.00	0.00	0.00	0.15	0.00	0.12	0.00	0.17	0.00	0.00	0.00	0.00	0.00	0.01	0.00	0.00	0.00	2.89	3.01	0.00	0.01	0.02
Thryssa mystax	0.00	0.00	0.00	0.00	0.00	0.00	0.01	0.00	0.23	0.00	0.00	0.01	1.87	0.00	0.01	0.00	0.00	0.00	0.00	0.00	0.03	0.02	0.00	4.18
Megalaspis cordyla	0.00	0.00	0.01	0.00	0.00	0.07	0.01	0.11	5.23	0.04	0.05	0.00	0.01	0.00	0.00	0.00	0.00	0.00	0.00	0.00	0.16	0.00	0.00	0.04
Cheilopogon doederleinii	0.00	0.00	0.00	0.00	0.00	0.00	0.00	0.00	0.00	0.00	0.00	0.00	0.00	0.00	0.00	0.00	0.00	0.00	0.00	0.00	0.00	5.47	0.03	0.02
Pennahia argentata	0.00	0.00	0.08	0.00	0.00	0.00	0.02	0.01	0.00	0.00	0.00	0.24	0.02	0.00	0.11	0.01	0.00	0.00	0.00	4.81	0.01	0.00	0.00	0.07
Sphyraena pinguis	0.00	0.00	0.01	0.00	0.01	0.18	0.01	0.10	1.67	0.08	0.01	0.00	0.02	0.01	0.00	0.00	0.01	0.00	0.00	0.05	2.65	0.00	0.01	0.02
Ctenopharyngodon idella	0.10	0.00	0.00	0.00	0.01	0.14	0.01	0.01	0.00	0.12	0.00	0.04	0.00	3.29	0.03	0.00	0.00	0.00	0.00	0.00	0.26	0.64	0.00	0.00
Larimichthys polyactis	0.00	0.00	1.48	0.01	0.00	0.00	0.00	0.00	0.00	0.01	0.07	0.00	0.04	0.01	0.61	0.05	0.02	0.01	0.00	0.05	0.17	1.66	0.03	0.01
Johnius trewavasae	0.00	0.00	0.00	0.00	0.00	0.00	0.01	0.00	0.00	0.00	0.00	0.33	0.03	0.00	0.31	0.01	0.00	0.00	0.00	3.52	0.00	0.00	0.00	0.02
Nemipterus nematophorus	0.00	0.00	0.00	0.04	0.00	0.00	0.00	0.04	3.13	0.00	0.02	0.00	0.00	0.00	0.00	0.00	0.00	0.00	0.00	0.00	0.00	0.00	0.00	0.00
Unclassified	13.05	13.89	0.57	93.66	0.49	12.56	0.08	0.20	0.36	0.94	63.65	0.24	35.13	0.94	49.61	96.28	0.30	42.62	0.95	0.76	18.93	81.47	99.27	38.35
Others	0.02	0.04	6.33	3.01	0.11	3.37	3.41	1.55	14.15	4.23	0.19	1.04	6.05	11.30	1.98	0.04	0.37	0.03	0.01	9.33	16.07	4.48	0.13	6.61

The results are expressed as the relative abundance of the species in the total number of sequence reads (%).

Table S3. The relative abundance of the top 10 species in SPPs detected by the 16S rRNA primer pairs was classified according to the IUCN red list status, fishing vulnerability, resilience, and price.

Species	General name	Common name	Chinese nane	Endangered level	Fishing vulnerability	Resilience	Price	SSP-01	SSP-02	SSP-03	SSP-04	SSP-05	SSP-06	SSP-07	SSP-08	SSP-09	SSP-10	SSP-11	SSP-12	SSP-13	SSP-14	SSP-15	SSP-16	SSP-17	SSP-18	SSP-19	SSP-20	SSP-21	SSP-22	SSP-23	SSP-24
Penaeus vannamei	Freshwater/Marine	Pacific white shrimp	南美白对虾	Data deficient/Not Evaluated	Low	High	Very high	40.34	7.81	25.63	38.88	17.71	36.93	7.24	1.87	38.05	21.92	27.55	4.16	38.94	50.29	92.76	75.46	7.76	29.14	25.82	32.20	68.24	98.57	8.14	18.11
Carassius auratus	Freshwater	Goldfish	金鱼	Least Concern	Moderate vulnerability	Medium	Unknown	44.33	31.16	15.37	1.00	71.87	21.72	24.35	88.73	3.61	67.17	1.94	73.86	13.55	14.62	0.93	0.44	10.78	7.64	59.26	0.49	3.64	0.09	0.10	11.10
Sus scrofa	Pork	Cinghiale	亚洲野猪	Least Concern				0.02	14.66	30.24	45.20	2.77	7.09	0.41	0.05	0.45	0.04	0.65	1.76	23.74	0.87	0.41	6.85	1.81	38.71	3.18	1.55	8.21	0.06	0.34	47.63
Gallus gallus	Chicken	Chicken	原鸡	Data deficient/Not Evaluated				6.16	0.01	0.01	0.01	0.00	0.00	0.00	0.00	0.00	0.00	0.00	0.01	0.07	0.00	0.00	1.24	58.96	1.83	2.63	2.24	1.20	0.36	88.14	0.13
Hypostomus plecostomus	Freshwater	Suckermouth catfish	清道夫鱼	Least Concern	Low vulnerability	Medium	Unknown	0.01	41.24	15.15	0.36	1.10	4.20	17.34	0.00	0.01	0.00	0.00	0.03	0.12	0.01	0.01	0.44	13.54	10.78	2.38	0.51	6.54	0.03	0.15	4.05
Penaeus monodon	Marine	Giant tiger prawn	斑节对虾	Data deficient/Not Evaluated	Low	High	Very high	0.01	0.19	0.60	0.67	0.34	24.47	0.00	0.29	1.43	1.70	68.10	0.01	0.09	0.08	2.57	14.25	0.08	0.16	0.30	0.00	0.00	0.00	0.00	0.00
Sparus aurata	Marine	Gilthead seabream	金头鲷	Least Concern	Low to moderate vulnerability	Medium	Very high	0.00	0.01	0.34	0.00	0.02	0.01	16.18	0.16	0.20	0.03	0.05	10.82	10.84	0.87	1.08	0.13	2.09	6.21	0.70	46.06	0.74	0.18	1.00	10.92
Nemipterus virgatus	Marine	Golden threadfin bream	金线鱼	Vulnerable	Low to moderate vulnerability	Medium	Very high	0.00	0.01	0.27	0.49	0.04	0.20	0.29	0.61	12.36	1.18	0.11	1.28	2.18	20.84	0.21	0.01	0.01	0.13	0.05	5.83	0.52	0.04	0.13	0.31
Priacanthus macracanthus	Marine	Red bigeye	大眼鲷	Least Concern	Low vulnerability	Medium	High	0.00	0.02	0.62	0.21	0.05	0.30	23.52	0.71	4.40	0.25	0.05	0.08	0.36	0.01	0.00	0.00	0.01	0.12	0.08	0.06	1.32	0.01	0.03	0.32
Sepia pharaonis	Cephalopod	Pharaoh cuttlefish	虎斑乌贼	Data deficient/Not Evaluated	Low vulnerability	High	High	0.00	0.00	4.33	7.64	0.17	0.32	0.02	0.00	0.00	0.00	0.00	0.00	0.00	0.00	0.00	0.00	0.00	0.00	0.00	0.01	0.00	0.00	0.00	0.00
Others								9.14	4.89	7.44	5.54	5.93	4.75	10.64	7.57	39.47	7.72	1.55	8.00	10.11	12.41	2.02	1.18	4.96	5.28	5.61	11.05	9.60	0.66	1.98	7.42

The relative abundance of the top 30 fish species in SPPs detected by the 12S rRNA primer pairs was classified according to the IUCN red list status, fishing vulnerability, resilience, and price.

Species	Freshwater/Marine	Common name	Chinese nane	Endangered level	Fishing vulnerability	Resilience	Price	SSP-01	SSP-02	SSP-03	SSP-04	SSP-05	SSP-06	SSP-07	SSP-08	SSP-09	SSP-10	SSP-11	SSP-12	SSP-13	SSP-14	SSP-15	SSP-16	SSP-17	SSP-18	SSP-19	SSP-20	SSP-21	SSP-22	SSP-23	SSP-24
Chitala ornata	Freshwater	Clown featherback	飾妝鎧甲弓背魚	Least Concern	High to very high vulnerability	Medium	Unknown	64.55	28.80	25.61	0.10	59.25	52.69	12.02	56.14	0.98	67.14	25.02	43.77	9.63	6.05	3.00	0.25	20.61	0.84	54.39	0.03	7.95	3.85	0.16	4.59
Hypophthalmichthys molitrix	Freshwater	Silver carp	鰱	Near Threatened	High vulnerability	Medium	Unknown	20.24	8.02	15.08	0.07	39.24	22.12	10.09	36.01	0.57	25.38	9.72	33.67	20.25	5.23	3.26	0.23	22.02	0.36	42.57	0.03	2.95	1.15	0.10	9.43
Pangasianodon hypophthalmus	Freshwater	Striped catfish	蘇氏鯰	Endangered	Very high vulnerability	Low	Unknown	1.15	48.78	1.61	0.02	0.01	0.05	23.11	0.01	0.04	0.04	0.00	0.01	0.42	0.03	0.11	2.82	48.13	56.09	1.70	0.02	25.69	0.09	0.11	10.66
Evynnis tumifrons	Marine	Yellowback seabream	赤鯮	Least Concern	Moderate vulnerability	Medium	Medium	0.00	0.00	14.01	0.08	0.00	0.00	14.99	0.09	0.02	0.00	0.31	17.16	21.97	0.46	22.14	0.26	7.42	0.00	0.03	65.77	0.16	0.10	0.01	20.27
Priacanthus arenatus	Marine	Atlantic bigeye	砂大眼鯛	Least Concern	Low vulnerability	High	High	0.00	0.00	0.03	0.00	0.09	3.87	33.18	2.81	0.04	0.14	0.00	0.00	0.01	0.04	0.00	0.00	0.01	0.00	0.00	0.01	9.17	0.00	0.04	0.08
Trichiurus japonicus	Marine	Largehead hairtail	日本带鱼	Not Evaluated	Moderate to high vulnerability	Medium	Unknown	0.00	0.00	30.07	0.16	0.09	2.02	0.01	0.87	0.01	1.30	0.01	0.00	0.01	0.00	0.00	0.00	0.01	0.00	0.00	1.41	10.79	0.00	0.06	0.07
Nemipterus randalli	Marine	Randall's threadfin bream	郎氏金線魚	Least Concern	Low vulnerability	Medium	High	0.00	0.00	0.00	0.00	0.01	0.32	0.03	0.26	0.03	0.03	0.00	0.07	0.00	22.10	0.10	0.01	0.00	0.01	0.00	5.39	1.48	0.01	0.01	0.09
Upeneus sulphureus	Marine/Freshwater	Sulphur goatfish	黃帶緋鯉	Least Concern	Low vulnerability	Medium	High	0.00	0.00	0.54	0.30	0.00	0.00	0.33	0.22	24.26	0.00	0.24	0.01	0.96	0.00	0.01	0.00	0.06	0.00	0.00	0.02	0.02	0.01	0.00	1.76
Nemipterus japonicus	Marine	Japanese threadfin bream	日本金线鱼	Least Concern	Low vulnerability	Medium	High	0.00	0.00	0.11	0.53	0.02	0.49	0.20	0.29	4.02	0.07	0.03	0.11	0.21	10.47	0.27	0.02	0.05	0.00	0.00	5.90	0.28	0.00	0.00	0.14
Cyprinus carpio	Freshwater	Common carp	鯉	Vulnerable	High vulnerability	Medium	Unknown	0.48	0.28	0.02	0.00	0.05	1.34	0.02	0.02	0.01	0.02	0.24	0.15	0.01	13.46	0.10	0.00	0.01	0.01	0.00	0.00	0.06	0.99	0.01	0.00
Hypophthalmichthys nobilis	Freshwater	Bighead carp	鱅	Data deficient	High vulnerability	Medium	Unknown	0.42	0.17	4.26	0.02	0.63	0.63	2.12	0.71	0.01	0.25	0.08	0.99	1.71	1.39	0.18	0.01	0.80	0.00	0.34	0.00	0.07	0.02	0.00	1.75
Oreochromis niloticus	Freshwater	Nile tilapia	尼罗罗非鱼	Least Concern	Low to moderate vulnerability	Medium	Unknown	0.00	0.00	0.01	0.00	0.00	0.00	0.02	0.00	0.00	0.01	0.00	0.07	0.02	15.55	0.11	0.00	0.07	0.01	0.00	0.00	0.01	0.01	0.00	0.00
Priacanthus macracanthus	Marine	Red bigeye	短尾大眼鯛	Least Concern	Low vulnerability	Medium	High	0.00	0.00	0.02	1.14	0.00	0.00	0.00	0.09	13.02	0.00	0.08	0.02	0.31	0.00	0.00	0.00	0.04	0.00	0.00	0.00	0.01	0.01	0.00	0.65
Priacanthus tayenus	Marine	Purple-spotted bigeye	長尾大眼鯛	Least Concern	Low vulnerability	High	High	0.00	0.00	0.00	0.40	0.00	0.00	0.00	0.10	11.02	0.00	0.06	0.00	0.01	0.00	0.00	0.00	0.01	0.00	0.00	0.00	0.00	0.00	0.00	0.02
Gadus morhua	Marine	Atlantic cod	大西洋鱈	Vulnerable	High to very high vulnerability	Medium	Medium	0.00	0.00	0.00	0.00	0.00	0.00	0.00	0.00	0.01	0.02	0.00	1.64	0.08	0.11	8.70	0.00	0.00	0.00	0.00	0.00	0.02	0.00	0.00	0.00
Ictalurus punctatus	Freshwater	Channel catfish	斑点叉尾鮰	Least Concern	High to very high vulnerability	Low	Unknown	0.00	0.00	0.00	0.00	0.00	0.00	0.00	0.00	0.00	0.00	0.00	0.05	0.00	9.50	0.06	0.00	0.00	0.00	0.00	0.00	0.00	0.01	0.00	0.00
Gadus chalcogrammus	Marine	Alaska pollock	狹鱈	Not Evaluated	High vulnerability	Low	Low	0.00	0.00	0.00	0.00	0.00	0.00	0.00	0.00	0.00	0.00	0.00	0.35	0.06	0.05	8.95	0.00	0.00	0.00	0.00	0.00	0.00	0.00	0.00	0.00
Sardinella fijiensis	Marine	Fiji sardinella	斐济小沙丁鱼	Least Concern	Low vulnerability	High	Medium	0.00	0.00	0.04	0.00	0.00	0.00	0.05	0.07	8.42	0.00	0.08	0.00	0.05	0.00	0.00	0.00	0.00	0.00	0.00	0.00	0.01	0.00	0.00	0.32
Pentaprion longimanus	Marine	Longfin mojarra	長臂鑽嘴魚	Least Concern	Low to moderate vulnerability	High	Medium	0.00	0.00	0.00	0.25	0.00	0.00	0.00	0.09	8.19	0.00	0.06	0.00	0.00	0.00	0.00	0.00	0.00	0.00	0.00	0.00	0.01	0.00	0.00	0.00
Nemipterus marginatus	Marine	Red filament threadfin bream	緣金線魚	Least Concern	Low vulnerability	High	High	0.00	0.00	0.09	0.19	0.00	0.00	0.27	0.05	4.57	0.00	0.03	0.01	1.10	0.01	0.35	0.00	0.04	0.00	0.00	0.00	0.01	0.00	0.00	0.85
Atrobucca nibe	Marine	Blackmouth croaker	黑姑鱼	Least Concern	High vulnerability	Medium	High	0.00	0.00	0.00	0.00	0.00	0.15	0.00	0.12	0.00	0.17	0.00	0.00	0.00	0.00	0.00	0.01	0.00	0.00	0.00	2.89	3.01	0.00	0.01	0.02
Thryssa mystax	Marine	Moustached thryssa	中颌棱鳀	Least Concern	Low vulnerability	High	Medium	0.00	0.00	0.00	0.00	0.00	0.00	0.01	0.00	0.23	0.00	0.00	0.01	1.87	0.00	0.01	0.00	0.00	0.00	0.00	0.00	0.03	0.02	0.00	4.18
Megalaspis cordyla	Marine	Torpedo scad	鐵甲	Least Concern	Low to moderate vulnerability	Medium	High	0.00	0.00	0.01	0.00	0.00	0.07	0.01	0.11	5.23	0.04	0.05	0.00	0.01	0.00	0.00	0.00	0.00	0.00	0.00	0.00	0.16	0.00	0.00	0.04
Cheilopogon doederleinii	Marine	Flying fish	細牙燕鰩魚	Not Evaluated	Low vulnerability	High	Medium	0.00	0.00	0.00	0.00	0.00	0.00	0.00	0.00	0.00	0.00	0.00	0.00	0.00	0.00	0.00	0.00	0.00	0.00	0.00	0.00	0.00	5.47	0.03	0.02
Pennahia argentata	Marine	Silver croaker	黃順	Least Concern	Low to moderate vulnerability	High	Very high	0.00	0.00	0.08	0.00	0.00	0.00	0.02	0.01	0.00	0.00	0.00	0.24	0.02	0.00	0.11	0.01	0.00	0.00	0.00	4.81	0.01	0.00	0.00	0.07
Sphyraena pinguis	Marine	Red barracuda	油魣	Not Evaluated	Moderate vulnerability	Medium	Medium	0.00	0.00	0.01	0.00	0.01	0.18	0.01	0.10	1.67	0.08	0.01	0.00	0.02	0.01	0.00	0.00	0.01	0.00	0.00	0.05	2.65	0.00	0.01	0.02
Ctenopharyngodon idella	Freshwater	Grass carp	草鱼	Least Concern	High to very high vulnerability	Low	Unknown	0.10	0.00	0.00	0.00	0.01	0.14	0.01	0.01	0.00	0.12	0.00	0.04	0.00	3.29	0.03	0.00	0.00	0.00	0.00	0.00	0.26	0.64	0.00	0.00
Larimichthys polyactis	Marine	Yellow croaker	小黄鱼	Least Concern	Low to moderate vulnerability	Medium	Very high	0.00	0.00	1.48	0.01	0.00	0.00	0.00	0.00	0.00	0.01	0.07	0.00	0.04	0.01	0.61	0.05	0.02	0.01	0.00	0.05	0.17	1.66	0.03	0.01
Johnius trewavasae	Marine	Trewavas croaker	屈氏叫姑魚	Least Concern	Low vulnerability	High	Medium	0.00	0.00	0.00	0.00	0.00	0.00	0.01	0.00	0.00	0.00	0.00	0.33	0.03	0.00	0.31	0.01	0.00	0.00	0.00	3.52	0.00	0.00	0.00	0.02
Nemipterus nematophorus	Marine	Doublewhip threadfin bream	双鞭线鰆鯛	Least Concern	Low vulnerability	High	High	0.00	0.00	0.00	0.04	0.00	0.00	0.00	0.04	3.13	0.00	0.02	0.00	0.00	0.00	0.00	0.00	0.00	0.00	0.00	0.00	0.00	0.00	0.00	0.00
Unclassified								13.05	13.89	0.57	93.66	0.49	12.56	0.08	0.20	0.36	0.94	63.65	0.24	35.13	0.94	49.61	96.28	0.30	42.62	0.95	0.76	18.93	81.47	99.27	38.35
Others								0.02	0.04	6.33	3.01	0.11	3.37	3.41	1.55	14.15	4.23	0.19	1.04	6.05	11.30	1.98	0.04	0.37	0.03	0.01	9.33	16.07	4.48	0.13	6.61

The results are expressed as the relative abundance of the species in the total number of sequence reads (%).
